# Variability of Flowering Sex and Its Effect on Agronomic Trait Expression in White Guinea Yam

**DOI:** 10.3389/fpls.2022.837951

**Published:** 2022-04-25

**Authors:** Kohtaro Iseki, Ryo Matsumoto, Olajumoke Olaleye, Motoki Shimizu, Asrat Asfaw

**Affiliations:** ^1^Japan International Research Center for Agricultural Sciences (JIRCAS), Tsukuba, Japan; ^2^International Institute of Tropical Agriculture (IITA), Ibadan, Nigeria; ^3^Iwate Biotechnology Research Center (IBRC), Kitakami, Japan

**Keywords:** dioecious species, *Dioscorea rotundata*, flowering, tuber yield, white guinea yam, sex phenotype

## Abstract

White Guinea yam (*Dioscorea rotundata*) is mainly a dioecious tuberous crop that produces flowers of varying sex phenotypes. Agronomic traits in Guinea yam differ according to the sex phenotype, but the precise interaction between the traits and sex phenotype is not clearly understood. This might be due to the high heterozygosity of yam where cultivars with different flowering sex have different genetic backgrounds, which mask the sole effect of sex phenotype on the agronomic traits. This study used F_1_-derived clonal progenies from a bi-parental cross to minimize the impact of different genetic backgrounds among the plants with different sex phenotypes. The impact of plant sex on agronomic traits, specifically tuber yield, was evaluated through field trials conducted for four years. The results showed that only plants with a female genotype exhibited varying sex phenotypes even within the clones of same accession grown in the same experimental field. The significant effects of sex genotype and phenotype on agronomic traits were detected. Our results revealed that the flowering date was delayed in the plants with female genotypes compared to male genotypes, even when compared only among the plants with male phenotypes. The flowering date is the most important reason for the sexual differences in tuber yield. A high tuber yield was obtained when plants with the female phenotype flowered before tuber enlargement. This result can be attributed to the fact that the low flowering intensity in female plants increases the availability of carbon resources for leaf development. Female plants also showed a large negative effect of late flowering on tuber yield owing to resource competition between flowering and tuber enlargement. These findings demonstrate the feasibility of yield improvement by controlling the flowering time, with a higher effectiveness achieved in female than in male plants.

## Introduction

White Guinea yam (*Dioscorea rotundata* Poir.) is a tuber crop widely cultivated in West Africa, accounting for more than 90% of the global yam production (Asiedu and Sartie, 2010). Yam is important for food security and income generation in this region, but the annual yield has been stagnant for decades and varies from year to year (FAOSTAT, 2022). Variability of tuber yield and yield-related traits has been observed even among plants of the same variety grown in the same environment (Cornet et al., 2014). Variation in phenotypic expression has led to small genetic gains in traditional breeding since variety selection has been based on field observation (Darkwa et al., 2020a). It also interrupts the precise prediction of genetic parameters, such as the dominance and additive effects of the traits used to evaluate genetic gains (Asfaw et al., 2021). The unstable phenotypic expression has been a significant challenge in yam breeding.

This study focused on the sex of yam flowers as one of the causes of the unstable phenotypic expression because the sex of flowers has been known to interact with tuber yield (Akoroda et al., 1984; Dansi et al., 1999). The yam is mainly a dioecious species, with male and female flowers on different plants. Moreover, plant-to-plant variability in flower sex expression is common in yam fields (Mondo et al., 2020). A better understanding of the relationship between flowering sex and agronomic traits might be critical for genetic improvement in yam breeding.

Dansi et al. (1999) surveyed 560 accessions of yam from the *D. rotundata–D. cayenensis* complex and found that the distribution of male and female accessions was regionally biased. The former had higher tuber numbers per plant and total tuber yield than the latter. In contrast, Akoroda et al. (1984) reported higher tuber yields in females than in male plants. They explained that early flowering of male plants restricted leaf development due to resource competition, which caused low tuber yield. Little research has been conducted on the variability of yam flowering relevant to agronomic trait performance.

The complexity of the effects of the sex of yam flowers is attributed to three factors. The first is the variable phenotypic expression of the sex of the flowers. A plant with female flowers frequently shows male and monoecious flowers depending on the growing environment (Hamadina et al., 2009). Phenotype needs to be monitored for several growing seasons (years) to detect the precise interactions between the sex phenotype and tuber yield because it might change every year, even in plants within the same accession. The second is the high heterozygosity of yam accessions. Previous studies have evaluated the interactions between the sex of the flowers and other agronomic traits using male and female accessions with diverse genetic backgrounds (Dansi et al., 1999). It is considered that the effect of the flowers’ sex on tuber yield and other related traits might be masked by different genetic backgrounds among cultivars or accessions with male or female phenotypes. The third is the unavailability of genetic information about sex determination in yam. The visually observed flower sex, the sex phenotype, would be genetically controlled by sex determination loci, the sex genotype, which is expressed differently according to the environment (Petit et al., 2020). Both sex phenotype and genotype should be considered for their effect on tuber yield. Currently, sex genotype has been estimated only visually.

This study aimed to clarify, systematically, the interaction between yam flowering sex and agronomic traits concerning tuber yield. We analyzed an F_1_ cross-population comprising 199 progenies to minimize the effect of differences in genetic background. The sex genotype was determined using DNA markers previously developed by Tamiru et al. (2017). The effect of sex genotype and phenotype on the agronomic traits was analyzed using the data obtained from 4 years of field cultivation.

## Materials and Methods

### Plant Materials and Growth Conditions

A total of 199 F_1_-derived clonal progenies from the bi-parental cross were cultivated for four years from 2015 to 2018 in the experimental fields of the International Institute of Tropical Agriculture (IITA), Ibadan, Nigeria (7° 29 N, 3° 54 E). The male parent (TDr97/00777) showed only male flowers, and an accession showing monoecious flowers, TDr04-219, was selected as the female parent to obtain not only male and female plants but also monoecious plants to analyze the effects of sex phenotypes on agronomic traits. The female parent had slightly higher tuber yield, later flowering, and later senescence but other agronomic traits were similar to those of the male parent. A total of 248 F_1_ seedlings were grown in 2013, and the tubers were obtained in 2014. Plants propagated from the same tuber were regarded to be of the same accession and used for further multiplication. During the propagation, 49 accessions were lost due to poor plant growth, and the remaining 199 accessions were used for the experiment. To avoid the effects of seed tuber size on plant growth (Iseki and Matsumoto, 2020), 100 g of tuber blocks (setts) with a skin surface where a shoot bud could emerge were equally cut from the center part of a normal-sized tuber weighing approximately 1–2 kg. The setts were treated with a fungicide and planted in plastic pots (12 cm in diameter and 10 cm in height) filled with sterilized topsoil (sandy loam soil of pH 7.6 containing 2.0 g kg^–1^ organic carbon, 0.40 g kg^–1^ nitrogen, and 3.8 mg kg^–1^ Bray-1 phosphate) for pre-sprouting. The planting dates were June 15, May 16, May 2, and April 25 in 2015, 2016, 2017, and 2018, respectively.

**FIGURE 1:**
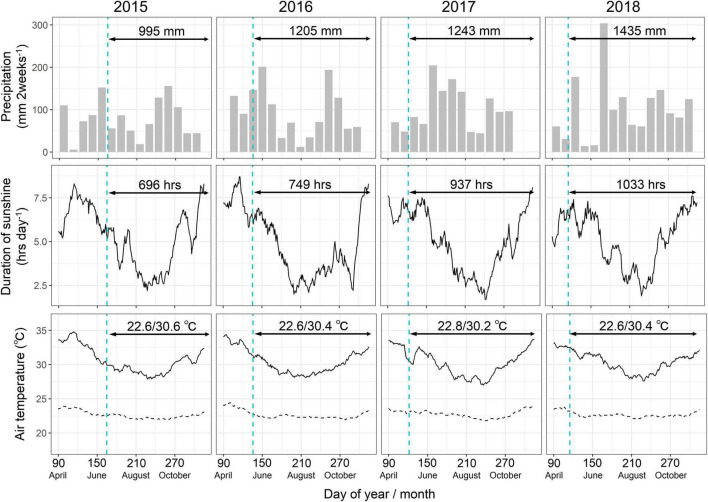
Meteorological conditions during the experimental period. The precipitation, duration of light, and air temperatures are separately shown for all the years. Arrows indicate the plant growth periods in each year. Numbers with the arrows represent total precipitation (mm), total duration of light (hours), and average maximum/minimum temperatures (°C) during the plant growth periods. Vertical dashed lines represent the planting date of each year.

After a month, plants with adequate sprouts were selected for each accession and transplanted with stakes at the top of 40-cm-high ridges prepared in the field. The distance between the ridges and between the plants on the ridges was 1 m, resulting in a plant density of 1 plant m^–2^. The field experiment was a randomized block design with three replications, each having one plant except the parents, which had five plants per replication. The total number of samples was 2,508, comprising 2,388 samples of 4 years from 199 accessions (one plant per plot and three replications) and 120 samples of 4 years from two parental accessions (five plants per plot and three replications). Weeding was performed manually when required, and fertilizers were not used in this study. The soil organic carbon, total nitrogen, and Bray-1 phosphate contents were 4.3, 0.39, and 3.1 mg kg^–1^, respectively. Before transplanting, the soil was plowed to ensure uniform soil conditions in the field. Meteorological data were obtained from a weather station located in IITA during the experimental period (Figure 1). The average rainfall pattern was bimodal with a short dry spell in early–mid-August. The total precipitation was lower in 2015 and higher in 2018 than the historical average of 1200 mm during the plant growth period of June–December at the experimental site. The rainfall in 2017 and 2018 was relatively low during the planting season and extremely high in the post-planting season, corresponding to late June–late July. The duration of sunshine was lower in 2015 than in 2018. The average maximum and minimum temperatures were not significantly different over the years, but the maximum temperatures around August–September tended to be lower in 2017 and 2018 than in the other years.

### Evaluation of Sex Phenotype and Agronomic Traits

**FIGURE 2:**
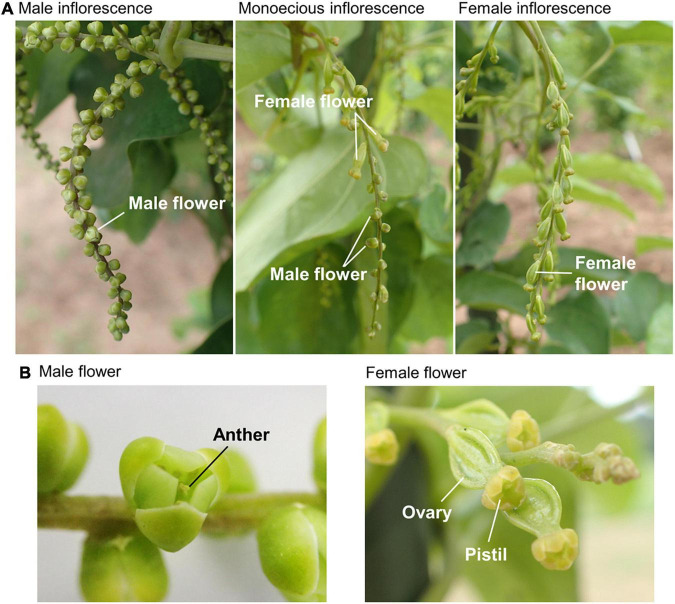
Sex phenotypes in *Dioscorea rotundata*. **(A)** Photos of male **(left)**, female **(right)**, and monoecious **(center)** inflorescences taken at the experimental field in IITA were shown. Male and female inflorescences consist only of male and female flowers, respectively. A plant of the first type monoecious has inflorescences with both male and female flowers. **(B)** Macro photos of the male **(left)** and female **(right)** flowers.

The sprouting dates were recorded for all the plants before transplanting. The number of days between planting and sprouting was used as an indicator of dormancy. Flowering date and sex phenotype were evaluated for each plant once a week from early July when flowering buds were initiated. Since many inflorescences are present in a single plant of *D. rotundata*, the flowering date was defined as the date of the emergence of the first inflorescence in a plant and was recorded as the day of the year (DOY). The sex phenotype was classified into three types: male, female, and monoecious (Figure 2). The plants that had only male inflorescence were recorded as “Male,” and those with only female inflorescences were recorded as “Female.” The “Monoecious” plants included two flowering types. The first type consisted of inflorescences having both male and female flowers, and the second type consisted of separate male and female inflorescences present on the same plant. Plants without an inflorescence throughout the growth period were recorded as “non-flowering.” Senescence was considered at the starting date for shoot senescence, which was defined as the date when 25% of the leaves in a plant withered; it was determined by visual observation and expressed using the DOY. At full-plant maturity in December, tubers were dug out, and the number of tubers per plant, size of each tuber (kg tuber^–1^), and total tuber yield per plant (kg plant^–1^) were recorded.

### Determination of the Sex Genotype

Sex genotype was determined using the DNA marker, sp16. The sp16 marker specifically amplifies the genomic region associated with female heterogametic (male = ZZ, female = ZW) sex determination (Tamiru et al., 2017). Leaf samples were collected from field-grown plants, immediately placed in liquid nitrogen, and then maintained at −80°C until lyophilization. Lyophilized leaves were sent to the Iwate Biotechnology Research Center, Japan, for DNA extraction and marker analysis. Total genomic DNA was extracted from the lyophilized leaf samples using a NucleoSpin Plant II Kit according to the manufacturer’s protocol (Macherey-Nagel GmbH & Co., Germany) with slight modifications. PCR amplification was performed for the sp16 marker and control actin gene fragment of *D. rotundata* in a 10 μL reaction mixture containing 10 ng genomic DNA, using 2 × EmeraldAmp MAX PCR Master Mix kit (Takara Bio Inc., Japan). The PCR conditions were as follows: 30 cycles of denaturation at 98°C for 10 s, annealing at 55°C for 30 s, and extension at 72°C for 1 min. All PCR products were electrophoresed on a 1.5% agarose gel.

### Statistical Analysis

The relationships between sex phenotype and other traits were analyzed by treating each replication as one sample because the sex phenotype was different for every plant, even within the same accession. The effects of sex phenotype, sex genotype, and experimental year on the agronomic traits were evaluated for 2,508 individuals using nested analysis of variance (ANOVA). The factor of sex phenotype was nested under the factor of sex genotype because the former was controlled by the later. The effect of accession was not considered in the analysis because it is closely related to the factor of sex genotype. The *F*-values and percentage of contribution (ρ) obtained in ANOVA were compared among the factors. Multiple comparison analysis by Tukey’s range test was separately performed for each year to detect significant differences in agronomic traits (dormancy, flowering date, senescence, tuber number, tuber size, and tuber yield) among sex phenotypes. Correlation between traits for each sex phenotype and experimental year was estimated using Pearson’s method. All analyses were performed using the statistical software R version 3.4.1 (R Core Team, 2018).

## Results

### Relationship Between Sex Phenotype and Sex Genotype

**FIGURE 3:**
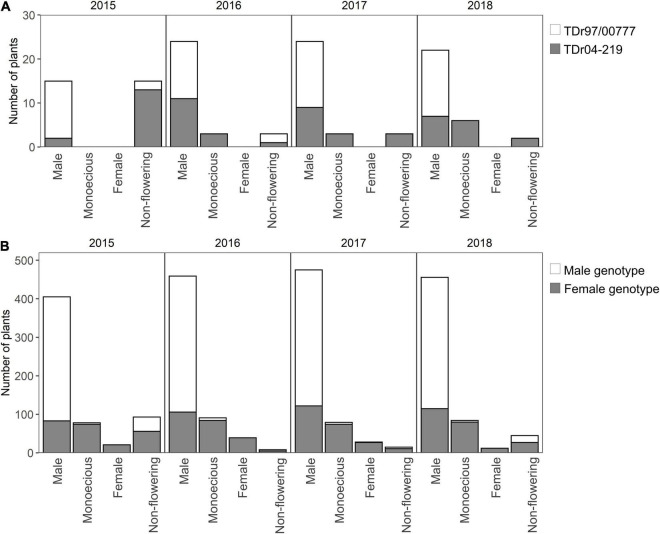
Distribution of sex phenotype in the parental plants and the cross population. The number of plants in each of the sex phenotype is separately shown for sex genotypes. **(A)** Sex phenotype distribution in the parents. A total of 15 samples were taken for each parent. **(B)** Sex phenotype distribution in the F_1_ population. The total numbers of plants of male and female genotypes were 363 (121 accessions × 3 replications) and 234 (78 accessions × 3 replications), respectively.

The male parent (TDr97/00777) only showed male phenotypes throughout the 4 years, although non-flowering plants were observed in 2015 and 2016 (Figure 3A). The female parent (TDr04-219) showed both male and monoecious phenotypes, among which more than 50% of the plants, except the non-flowering plants, were determined as “Male.” However, the female phenotype was not observed in TDr04-219 throughout the 4 years. All the monoecious plants of the parents showed monoecious inflorescences with both male and female flowers (the first type of monoecious).

Among the 199 accessions of the F_1_ population, the ratio of male and female genotype was distorted from the theoretical expectation of 1:1 and 121 accessions, accounting for 60%, had male genotype, and 78 accessions had female genotype. The sex genotype and observed sex phenotype of each accession are summarized in Supplementary Table 1. Among the plants with male genotype, on an average, more than 90% showed male phenotype, and the other 10% were “Monoecious” or “Non-flowering” (Figure 3B); the monoecious phenotype was only observed in two accessions of MP2-142 and MP2-169, and no female phenotype was found throughout the 4 years. Among the plants with female genotype, 5%–17% were female, and the remaining were “Male” or “Monoecious.” Throughout the 4 years, most of the monoecious plants had both male and female flowers in an inflorescence (the first type), and only two plants had separate male and female inflorescences in a plant (the second type). The accessions MP2-098 and MP2-221 with female genotype and MP2-190 with male genotype rarely flowered. Additionally, out of the 12 plants (3 plants per year), only 2–4 plants flowered during the 4 years. Although many plants did not flower in 2015, the distribution ratio of the sex phenotypes among the F_1_ population tended to be similar throughout the 4 years.

### Effects of Sex Types on Agronomic Traits

Among all the tested agronomic traits, the factor of year had the largest contribution that accounted for 17.7–49.9% of the total trait variation (Table 1). Sex genotype had especially major effects on the flowering date, which accounted for 7.9% of the total variation. Additionally, the effect of the sex genotype on the flowering date was confirmed when compared with the plants only of the male phenotype. The plants with female genotype showed significantly later flowering than those with male genotype (Table 2). However, the effects of sex genotype on other traits were unclear. The effect of sex phenotype was shown as the interaction with sex genotype in the nested ANOVA. It had significant effects for all the tested agronomic traits. The greatest impact was observed for flowering date and tuber yield, which accounted for 3.3 and 3.2% of the total variation, respectively. Except the flowering date, the contribution of sex phenotype was always larger than that of sex genotype. Significant interactions among the factors were also detected, but almost all the contributions were lower than those of the year or sex phenotype.

**TABLE 1:** *F*-values and percentage of contribution (ρ) of the factors obtained by the nested ANOVA for the variation of agronomic traits.

	Dormancy			Flowering date			Senescence		
	Degree of freedom	*F*-value	ρ (%)	Degree of freedom	*F*-value	ρ (%)	Degree of freedom	*F*-value	ρ (%)
Year	3	273**	24.2	3	1,019**	49.9	3	174**	17.7
Sex genotype	1	9**	0.2	1	486**	7.9	1	0 ^ns^	0.0
Sex genotype × Sex phenotype	6	11**	1.8	4	51**	3.3	6	4**	0.7
Year × Sex genotype	3	3*	0.2	3	13**	0.6	3	0 ^ns^	0.0
Year × Sex genotype × Sex phenotype	14	4**	1.1	9	4**	0.4	14	1 ^ns^	0.1

	**Tuber number**			**Tuber weight**			**Tuber yield**		
	**Degree of freedom**	***F*-value**	**ρ (%)**	**Degree of freedom**	***F*-value**	**ρ (%)**	**Degree of freedom**	***F*-value**	**ρ (%)**

Year	3	190**	18.5	3	183**	17.8	3	345**	28.6
Sex genotype	1	0^ns^	0.0	1	6*	0.2	1	4*	0.1
Sex genotype × Sex phenotype	6	8**	1.4	6	12**	2.1	6	20**	3.2
Year × Sex genotype	3	2 ^ns^	0.1	3	2^ns^	0.1	3	1 ^ns^	0.0
Year × Sex genotype × Sex phenotype	13	3**	1.1	13	4**	1.3	13	4**	1.1

The factor of sex phenotype was nested under the factor of sex genotype. **P < 0.01, *P < 0.05, and ns: not significant.

**TABLE 2:** Trait comparison between the sex genotypes in the plants with male phenotype.

Trait	Year	Male genotype mean	Female genotype mean	*P* value
Dormancy (days to sprouting)	2015	30.1	31.3	0.09^ns^
	2016	25.8	27.2	0.01*
	2017	26.2	28.5	< 0.01**
	2018	20.5	21.2	0.27^ns^
Flowering date (day of year)	2015	224	234	< 0.01**
	2016	192	202	< 0.01**
	2017	207	214	< 0.01**
	2018	198	203	< 0.01**
Senescence (day of year)	2015	313	312	0.45^ns^
	2016	302	303	0.79^ns^
	2017	304	306	0.02*
	2018	309	310	0.36^ns^
Tuber number (tubers plant^–1^)	2015	2.06	2.45	0.01*
	2016	1.17	1.09	0.10^ns^
	2017	1.36	1.36	0.96^ns^
	2018	1.26	1.10	< 0.01**
Tuber size (kg tuber^–1^)	2015	1.26	1.06	0.03*
	2016	1.68	1.81	0.05^ns^
	2017	1.05	1.07	0.70^ns^
	2018	0.98	1.17	< 0.01**
Tuber yield (kg plant^–1^)	2015	2.00	1.92	0.34^ns^
	2016	1.81	1.89	0.17^ns^
	2017	1.18	1.21	0.44^ns^
	2018	1.12	1.23	0.02*

**P < 0.01, *P < 0.05, and ^ns^: not significant.

**FIGURE 4:**
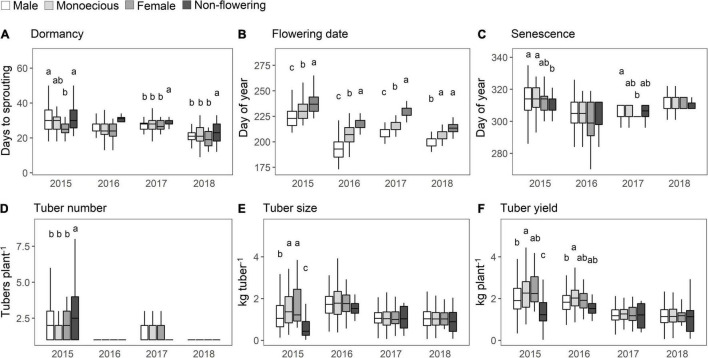
Distribution of agronomic traits in each of the sex phenotypes. **(A)** Dormancy. **(B)** Flowering date. **(C)** Plant senescence. **(D)** Tuber number. **(E)** Tuber size. **(F)** Tuber yield. Trait values in the F_1_ population are shown in the boxplot. The horizontal lines in the boxes are the median values. The height of the box is equal to the interquartile distance, indicating the distribution for 50% of the data. Approximately 99% of the data fall between the top and bottom of the lines extending from the box. Bars with different letters show significant difference at the *P* < 0.05. The monoecious phenotype includes both the first and second types.

The distribution of the values of the agronomic traits was compared among the sex phenotypes and years. On average, non-flowering plants took longer to sprout than the flowering plants (Figure 4A), and significant differences were observed in 2017 and 2018. The flowering date for the “Male” phenotype was the earliest, followed by the “Monoecious” phenotype (Figure 4B); this finding was consistent during the 4 years. The delayed planting by approximately one month in 2015 resulted in a corresponding delay in flowering and senescence (Figure 4C) compared to the other years. The male plants showed delayed senescence in 2015 and 2017, unlike in the other years. There was no difference in tuber number based on the sex phenotype throughout the study period (Figure 4D). On average, “Monoecious” and “Female” phenotypes showed high values for tuber size and yield in 2015 and 2016 (Figures 4E,F). However, no sex differences were observed in 2017 and 2018, when the size and yield were lower than those in the previous two years.

### Correlations Among the Agronomic Traits

**FIGURE 5:**
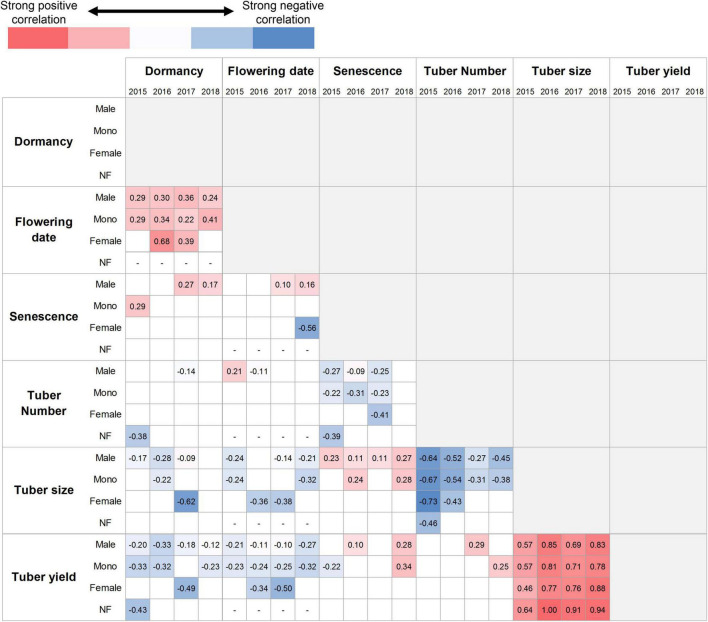
Trait correlations among the F_1_ population. Pearson’s correlation coefficient with statistical significance at *P* < 0.05 level is separately shown for sex phenotypes and years. “Mono” and “NF” denote the monoecious and non-flowering plants, respectively. The monoecious phenotype includes both the first and second types. The empty boxes indicate that the relationship is not significant. The sex phenotypes and years are indicated in rows and columns, respectively. The red and blue colors represent the positive and negative relationships; the deeper the color, the stronger the correlation. The number of plants analyzed in each box corresponds to Figure 3B.

Correlations between the traits were assessed separately for each sex phenotype and year, as shown in Figure 5. A significant correlation was rarely observed in the female and non-flowering plants because detecting significance depended on the number of samples. Dormancy was positively correlated to flowering date. Plants with later sprouting showed later flowering. The positive relationships between tuber size and tuber yield indicated that the plants with larger tuber sizes had more tuber yields. These relationships were consistent with the sex phenotypes and years. Senescence was also positively correlated with the tuber size, indicating that late senescence resulted in larger tubers; however, this relationship was observed only in male and monoecious plants, and the correlation coefficients were low. Negative correlations were found between the following pairs: senescence–tuber number, tuber size–tuber number, dormancy–tuber yield, and flowering date–tuber yield. These relationships were found in more than half of the cases among the combinations of sex phenotypes and years. The last two combinations indicated that late sprouting and flowering resulted in lower tuber yields. A similar relationship was partially observed for tuber size.

**FIGURE 6:**
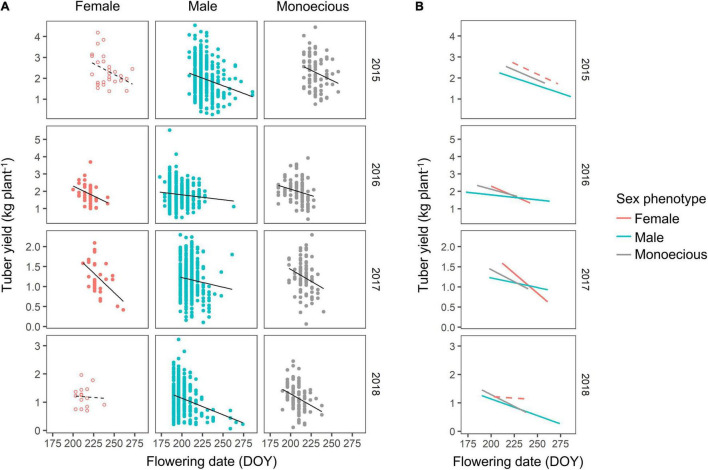
Correlation between flowering date and tuber yield. **(A)** The relationship is separately shown with a regression line for each of the sex phenotypes and years. The open circles with dashed regression line indicate that the correlation was not statistically significant at *P* < 0.05. The correlation coefficient and significance are the same as indicated in Figure 5. **(B)** The regression line of different sex phenotypes is compared.

The relationship between the flowering date and tuber yield was further analyzed (Figure 6A). Although there were large variations in tuber yield, the slopes of the correlations were different for the sex phenotypes. Plants with female phenotypes tended to show more negative slopes than other sex phenotypes (Figure 6B). The tendency of high tuber yield in female plants was observed when the flowering occurred during early August (DOY 210–225), but the yield differences among the sex phenotypes diminished when the flowering occurred after late August (> DOY 240).

## Discussion

### Relationship Between Sex Genotype and Sex Phenotype

The lability of the sex phenotype of TDr01-219 and the female genotypes in the F_1_ population (Figure 3) could be explained by the heterogametic sex-determination system in yam in which male and female plants have ZZ and ZW sex-determination loci, respectively (Tamiru et al., 2017). The female locus (W) suppresses the male locus (Z) expression, thus causing the expression of the female phenotype. Similar sex determination systems have been observed in several species. In *Melandrium album*, which has a female-suppressing sex-determination system, hypomethylation treatment reduced female suppression and increased the formation of female flowers in plants with male genotypes (Janoušek et al., 1996). This observation indicates that the expression of sex phenotype is controlled by hypomethylation, which is affected by the environmental conditions such as air temperature, soil water content, and light through changes in microRNA expression (Song et al., 2019). In yam, the sex phenotypes of female genotype plants varied even among the clone plants of the same accession growing in the same year. This finding suggests that the sex phenotype might be affected by differences in the microenvironments such as soil water conditions, which vary among the replication blocks within a field. Light conditions are considered another factor of sex lability (Varga and Kytöviita, 2016). The light conditions would be different for the plants inside and near the border or on the east and west sides of the field; such differences in light conditions affect the sex phenotype (Buide et al., 2018). Hence, sex phenotype in yam varies easily owing to slight environmental differences even within the same experimental field, making the sex determination of each accession and variety difficult.

The number of non-flowering plants was distinguished in 2015, and most of the non-flowering plants belonged to female genotype. This result might be attributed to the lower solar radiation in 2015, especially until 2 months after transplanting before flower initiation. Hamadina et al. (2009) reported that the flowering rate decreased in male plants under low temperature and low solar radiation conditions. In contrast, flowering in female plants was not affected by the environment. This finding was inconsistent with our result that most non-flowering plants were of female genotype; even among male genotype, the number of non-flowering plants was highest in 2015, but it was still less than that in female genotype. A reason for this inconsistency is that the previous study results were obtained from only two accessions, one male and one female; thus, differences in the genetic background could not be considered. Our results were obtained using 199 accessions of F_1_ population having similar genetic backgrounds, and indicated that the flowering rate of both male and female genotypes could be affected by the environment.

The high rate of non-flowering plants among female genotype partly explains the high ratio of male plants among the genetic resources. Girma et al. (2019) surveyed the flowering sex of 1,938 *D. rotundata* accessions and found that 85% were either male or non-flowering. This is primarily because female genotype shows both male and female phenotypes while male genotype only shows the male phenotype. On the contrary, yam plants grown from seeds showed a higher ratio of female plants than those derived from continuous vegetative propagation (Sadik and Okereke, 1975). This indicates that the ratio of female plants changes during continuous vegetative propagation in a population. In this study, the non-flowering plants, most of which had female genotype, showed smaller tuber sizes and lower tuber yields than the flowering plants. Low tuber yield enhances the risk of genotype loss owing to tuber rot during storage (Coursey, 1967) and, thus, might decrease the ratio of female-genotyped plants among the genetic resources over continuous vegetative propagation for years.

The lower tuber yield of non-flowering plants with female genotype also explained the distortion of male and female genotype ratios in the F_1_ population. More than 60% of the plants in the F_1_ population had a male genotype, which was higher than the theoretical 1:1 segregation expected in F_1_ seedlings derived from heterogametic female. This is because non-flowering plants with female genotypes might be diminished due to small tuber sizes and poor growth. A total of 49 accessions of the 248 F_1_ plants were lost through the propagation process, and most of them were expected to have female genotype.

Most of the plants with male genotype showed a stable male phenotype than the plants with female genotype, except for the two accessions MP2-142 and MP2-169, which showed monoecious flowers throughout the 4 years. A similar result was also observed by Tamiru et al. (2017), where some of the accessions with male genotype, determined using the sp16 DNA marker, showed monoecious flowering. A possible explanation for these results could be the suppression of the male phenotype by heritable autosomal activation of the female-derived genomic imprinting mechanisms (Janoušek et al., 1996); however, this mechanism has not yet been confirmed in yam species.

### Relationship Between Sex Phenotype and Agronomic Traits

The significant effects of sex phenotype on agronomic traits account for the inconsistency between the classification of genetic resources based on genotypic and phenotypic variation (Darkwa et al., 2020b). The sex of the flower varied over the years; this result explains the difficulty in evaluating sex phenotype in traditional breeding of yam. In addition, because the factor of year had the greatest effect, phenotype evaluation for only 1 or 2 years is not reliable. Continuous phenotyping is required for several years for precise evaluation of the agronomic traits. Furthermore, agronomic traits should be analyzed considering the effects of sex phenotype and genotype.

The meteorological conditions during the early growth periods primarily cause the yearly yield variation. According to multi-environmental field trials (Otoo and Asiedu, 2008), high tuber yields have been obtained in areas with 1,000–1,200 mm of annual precipitation, above which the tuber yield decreased. This finding indicates that excessive rainfall reduces the tuber yield. In 2017 and 2018, the annual precipitation was relatively high because of the high rainfall during the short dry spell in early August, resulting in low tuber yield.

Although the yearly effect explained more than 17% of the total trait variation, the sex phenotype still had a greater effect on the variations of most agronomic traits compared with that of sex genotype (Table 1). This result indicates that the agronomic traits vary according to the sex phenotype, which might be affected by the microenvironment within the field, even among the clone plants growing in the same field in the same year.

The flowering date is most affected by the sex phenotype and genotype (Figure 4). The plants with the male phenotype flowered earlier than those with the female phenotype. Our study demonstrated that flowering occurs earlier in plants with male genotype than those with female genotype, even when compared among the plants with the male phenotype (Table 2). This phenomenon has been confirmed through empirical observations and several studies (Akoroda et al., 1984). Girma et al. (2019) considered the genes related to photoperiod sensitivity and flowering as sex determination genes. Common mechanisms involving microRNAs could explain the interaction between sex phenotype and flowering date. In hemp (*Cannabis sativa*), a dioecious plant with a male-suppressing sex determination system similar to the yam, microRNA expression regulates the balance of phytohormones affecting both sex determination and flowering date (Petit et al., 2020). Since sex determination is regulated by a balance of phytohormones in many crops, a similar mechanism can be expected in yam (Adam et al., 2011; Chen et al., 2017; West and Golenberg, 2018).

However, the flowering date was also determined by the dormancy. Earlier flowering was brought by earlier sprouting (Figure 6). Male plants still had earlier flowering date when compared with the plants of same values in days to sprouting (Supplementary Figure 1). Therefore, actual flowering date for each plant was determined by the factors of sex genotype, sex phenotype, and sprouting, but it was more strongly associated with plant sex than sprouting. Tuber sprouting depends on environmental conditions, such as temperature and air humidity, during tuber storage (Osunde and Orhevba, 2009). It might affect the carbohydrate metabolism at sprouting (Muthukumarasamy and Panneerselvam, 2000). This implies that flowering date can be controlled through the manipulation of sprouting date using plant growth regulators (Shiwachi et al., 2003).

The tuber number, size, and yield were significantly affected by the sex phenotype and accounted for 1.4%–3.2% of the total variation (Table 1). The tendency of large tuber size and high tuber yield in plants with the female phenotype were distinguished in 2015 and 2016 (Figure 4). Akoroda et al. (1984) hypothesized that the late flowering in female plants avoided resource competition with leaf development. Thus, the plant biomass increases during the early growth period before flowering and results in a high tuber yield (Iseki and Matsumoto, 2020).

However, the above hypothesis could not fully explain our results. First, the trait correlation demonstrated in Figure 5 reveals that the flowering date and tuber yield are negatively correlated among the plants with the same sex phenotype, suggesting that the plants with later flowering have lower tuber yield, although the plants with late flowering could ensure leaf development before flowering. Second, the tuber size and tuber yield in 2017 and 2018 were similar, although the flowering date was significantly different among the sex phenotypes. Third, the tuber size and tuber yield of non-flowering plants tended to be lower than those of flowering plants, although there might be no resource competition between leaf development and flowering. Therefore, other mechanisms are expected to be involved in the higher tuber yield of female plants.

The female plants had a higher tuber yield even when the tuber yield was compared among the plants with the same flowering date (Figure 6). This result could be explained by the low flowering intensity of females that also reduced resource competition. Typically, flowering intensity is lower in female plants than in male plants (Hamadina et al., 2009). Therefore, the amount of carbon resources utilized for female flowering is less than that for male flowering, and thus, more carbon resources are available for leaf development in female plants.

In addition, the negative relationship between flowering date and tuber yield is thought to be caused by resource competition with tuber enlargement. Normally, tuber enlargement starts in late August (around DOY 240) at the experimental site (Vaillant et al., 2005), after which leaf development is retarded due to resource competition (Marcos et al., 2011; Iseki et al., 2021). Therefore, late-flowering occurring after late August restricts the utilization of carbon resources for tuber enlargement. This competition would become more severe in female plants that have a later flowering date than the plants of other sex phenotypes. This finding explains the large negative slope in the correlation between flowering date and tuber yield in female plants (Figure 6). The tuber yield is determined by the resource allocation between leaf development, flowering, and tuber enlargement, each of which consumes carbon resources. Late flowering can help avoid resource competition with leaf development; however, it causes resource competition with tuber enlargement when flowering occurs after the start of tuber enlargement.

## Conclusion

Our results indicated that tuber yield increased regardless of the sex phenotype when the flowering date was regulated to fall into the lag period between the early leaf developmental stage and the start of tuber enlargement. This strategy avoids the resource competition of flowering with leaf development or tuber enlargement. A larger yield increase is expected in the female plants because of their low flowering intensity, which implies that more carbon resources would be allocated for tuber enlargement. Because the sex phenotype varies with the surrounding environment in plants with female genotype, artificial control of sex phenotype would be possible for yield improvement of female-genotyped varieties and could be achieved by appropriate field management, such as soil water control and arrangement of plant light interception to maintain good culture conditions. In addition, flowering date would be possible to control by manipulation of sprouting date to avoid resource competition. These findings are believed to be applicable not only for the F_1_ population used in this study but also for other yam materials because the late flowering in female plants has been generally observed in wide genetic resources (Akoroda et al., 1984; Hamadina et al., 2009). However, the resource allocation does not completely explain the interaction between the flowering sex and tuber yield. In particular, this study could not elucidate the reason for lower tuber yield of non-flowering plants than that of the flowering plants. Further studies are needed to elucidate the mechanisms underlying the relationships between sex phenotype, flowering date, and tuber yield.

## Data Availability Statement

The original contributions presented in the study are included in the article/Supplementary Material, further inquiries can be directed to the corresponding author/s.

## Author Contributions

KI: conceptualization, data curation, methodology, and writing – original draft. RM: conceptualization, data curation, methodology, and writing – review and editing. OO and MS: data curation, methodology, and writing – review and editing. AA: conceptualization, writing – review and editing, and supervision.

## Conflict of Interest

The authors declare that the research was conducted in the absence of any commercial or financial relationships that could be construed as a potential conflict of interest.

## Publisher’s Note

All claims expressed in this article are solely those of the authors and do not necessarily represent those of their affiliated organizations, or those of the publisher, the editors and the reviewers. Any product that may be evaluated in this article, or claim that may be made by its manufacturer, is not guaranteed or endorsed by the publisher.
