# Supplementary material for: Variability of Flowering Sex and Its Effect on Agronomic Trait Expression in White Guinea Yam
Source: Front Plant Sci. 2022 Apr 25;13:837951. doi: 10.3389/fpls.2022.837951 (PMC9083005; doi:10.3389/fpls.2022.837951)
Supplement: Supplementary file 1 [file Table_1.DOCX]

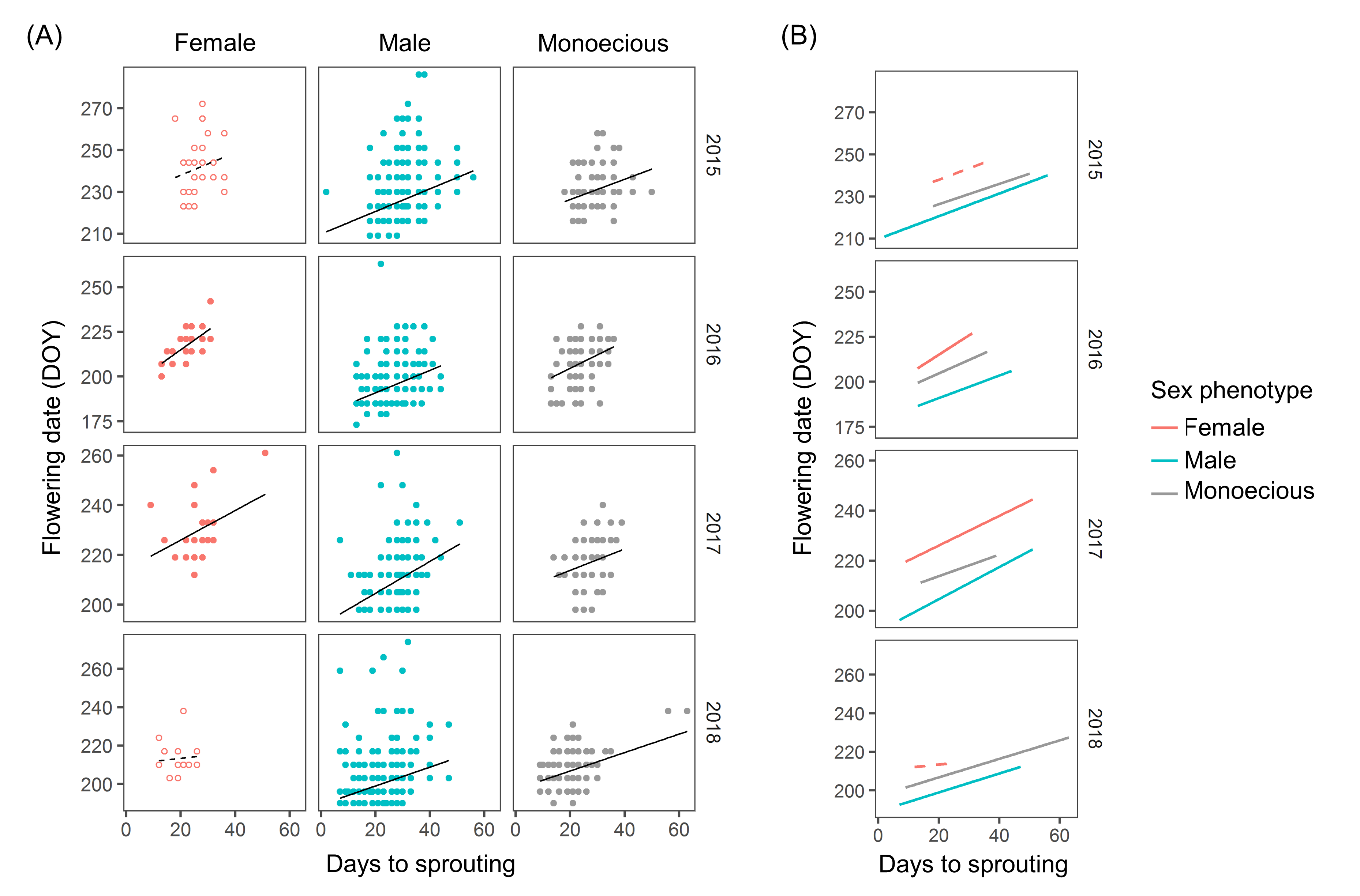


**Supplemental Figure S1.** Correlation between dormancy (days to sprouting) and flowering date. (A) The relationship is separately shown with a regression line for each of the sex phenotypes and years. The open circles with dashed regression line indicate that the correlation was not statistically significant at P <0.05. The correlation coefficient and significance are the same as indicated in Fig. 5. (B) The regression line of different sex phenotypes is compared.
